# Effect of IL-1β on NSCLC-Derived Small Extracellular Vesicles as Actors in Mediating Cancer Progression and Evading Immune System

**DOI:** 10.3390/ijms26146825

**Published:** 2025-07-16

**Authors:** Hamid Heydari Sheikhhossein, Luisa Amato, Viviana De Rosa, Caterina De Rosa, Annalisa Ariano, Sabrina Critelli, Daniela Omodei, Valeria Nele, Concetta Tuccillo, Paola Franco, Giovanni N. Roviello, Rosa Camerlingo, Adriano Piattelli, Giovanni Vicidomini, Floriana Morgillo, Giuseppe De Rosa, Maria Patrizia Stoppelli, Carminia Maria Della Corte, Natalia Di Pietro, Francesca Iommelli

**Affiliations:** 1 Department of Medical, Oral and Biotechnological Sciences, University “G. d’Annunzio” of Chieti-Pescara, 66100 Chieti, Italy; hamid.heydari@unich.it (H.H.S.); natalia.dipietro@unich.it (N.D.P.); 2 Department of Precision Medicine, University of Campania Luigi Vanvitelli, 80131 Naples, Italycaterina.derosa1@unicampania.it (C.D.R.); annalisa.ariano9826@gmail.com (A.A.); concetta.tuccillo@unicampania.it (C.T.); floriana.morgillo@unicampania.it (F.M.); carminiamaria.dellacorte@unicampania.it (C.M.D.C.); 3 Institute of Biostructures and Bioimaging, National Research Council, 80145 Naples, Italy; sabrinacritelli15@gmail.com (S.C.); daniela.omodei@ibb.cnr.it (D.O.); giovanni.roviello@cnr.it (G.N.R.); 4 Department of Pharmacy, University of Naples Federico II, 80131 Naples, Italy; valeria.nele@unina.it (V.N.); gderosa@unina.it (G.D.R.); 5 Institute of Genetics and Biophysics Adriano Buzzati Traverso, National Research Council, 80131 Naples, Italy; paola.franco@igb.cnr.it (P.F.); mpatrizia.stoppelli@igb.cnr.it (M.P.S.); 6 Department of Cell Biology and Biotherapy, Istituto Nazionale Tumori—IRCCS—Fondazione G. Pascale, 80131 Naples, Italy; r.camerlingo@istitutotumori.na.it; 7 School of Dentistry, Saint Camillus International University of Health and Medical Sciences, 00131 Rome, Italy; apiattelli51@gmail.com; 8 Facultad de Medicina, UCAM Universidad Católica San Antonio de Murcia, 30107 Murcia, Spain; 9 Department of Translational Medical Sciences, University of Campania Luigi Vanvitelli, 80131 Naples, Italy; giovanni.vicidomini@unicampania.it

**Keywords:** NSCLCs, tumor-derived small extracellular vesicles (TEVs), IL-1β, cancer progression, EMT, immune suppression

## Abstract

Background: Increased IL-1β levels may promote carcinogenesis and metastasis by affecting tumor biology and the tumor microenvironment (TME). In this context, extracellular vesicles (EVs) play a key role in cell-to-cell communication, thus modulating the TME and immune response. Here, we aimed to test whether tumor-derived small EVs (TEVs) isolated from sensitive and osimertinib-resistant (OR) non-small-cell lung cancer (NSCLC) cells may promote EMT via fibronectin binding to α5β1 integrin as well as suppress the immune system and if these effects may be favored by IL-1β. Methods: TEVs were isolated from control, OR, and IL-1β-stimulated NSCLC cells. Expressions of fibronectin and PD-L1 were screened in TEVs and the mRNA levels of vimentin and SMAD3 were also assessed in cancer cells after TEV co-culturing. Furthermore, to detect the effect on immune cells, we co-cultured TEVs with lung cancer patients’ peripheral blood mononuclear cells (PBMCs). Results: TEVs were positive for fibronectin and the highest protein levels were found in TEVs obtained from the OR and IL-1β-stimulated cells. TEV-mediated activation of α5β1 signaling led to the upregulation of vimentin and SMAD3 mRNA in NSCLC cells and stimulated cell migration. EVs also increased PD-1, CTLA-4, FOXP3, TNF-α, IL-12, and INF-γ mRNA in lung cancer patients’ immune cells. Conclusions: Our findings indicate that TEVs promote EMT in NSCLC cells by the activation of the fibronectin–α5β1 axis. Finally, IL-1β stimulation induces TEV release with biological properties similar to OR TEVs, thus leading to cancer invasion and immune suppression and suggesting that inflammation can promote tumor spreading.

## 1. Introduction

The tumor microenvironment (TME) represents a primary element in orchestrating cancer growth and progression and high levels of inflammatory cytokines may promote such programs. Inflammation may affect cancer biology and modulate the release of several cellular components in the extracellular space, thus favoring the establishment of a functional crosstalk between the tumor and its microenvironment. Secretion of extracellular vesicles (EVs) from different cell types constitutes a critical process in cell-to-cell communication, thus facilitating the transfer of bioactive cargo to recipient cells in the TME [1]. These EVs are composed of a phospholipid bilayer and can be released by various cell types, both damaged and healthy [2], and in several biological fluids, ranging from blood [3,4], cerebrospinal fluid [5], milk [6,7,8], amniotic fluid [9,10], urine [11,12,13], and semen [14]. EVs are categorized into three primary subtypes based on their characteristics (size, density gradient, and biogenesis): apoptotic bodies (ApoEVs), microvesicles (MVs), and exosomes (EXs). ApoEVs are vesicles released by dying cells while MVs bud from the plasma membrane. EXs are smaller than ApoEVs and MVs, with a diameter of approximately 30–150 nm, and are released through the endosomal pathway via the formation of multivesicular bodies (MVBs) [15,16,17,18]. In particular, EXs are classified as small EVs (sEVs) and although they have been identified as a main entity in such vesicle subtype [19], currently, the International Society for Extracellular Vesicles (ISEV) updated its ‘Minimal Information for Studies of Extracellular Vesicles’ (MISEV) and reported that sEVs represent a heterogeneous mixture of vesicles, including EXs and small ectosomes [16]. The overlapping size distributions and shared surface markers of the different sEV subtypes make reliable discrimination between them challenging. Specifically, sEVs carry several bioactive molecules such as nucleic acids, lipids, proteins, and carbohydrates and they may modulate the functions of recipient cells by both plasma membrane fusion and through the engagement of membrane receptors. Currently, it is well established that tumor-derived extracellular vesicles have a relevant role in cancer progression by acting in an autocrine or paracrine manner, thus modulating the biological properties of both cancer cells from which they are released and other different cells of the TME and immune system [20]. In this context, sEVs, such as exosomes, have been shown to promote the acquisition of an epithelial-to-mesenchymal transition (EMT) phenotype in fibroblasts and to enhance cancer cell survival in the circulatory system by suppressing immune surveillance [21,22,23,24]. In particular, the content and biological properties of EVs are influenced by environmental conditions, drug-induced injury, and stress stimuli such as chronic inflammation, all of which are associated with tumor progression and immune evasion [25,26,27]. In agreement with these observations, it is widely demonstrated that inflammatory cells were abundant in tumor biopsies and that inflammatory cytokines along with chemokines are key drivers of cancer-promoting inflammation [28]. Among the various pro-inflammatory cytokines, Interleukin-1 beta (IL-1β) plays a central role in linking inflammation to cancer [29,30], due to its elevated levels in a wide range of malignancies [31,32]. IL-1β contributes significantly to cancer development and progression by promoting uncontrolled cell proliferation, facilitating immune evasion, and supporting metastatic spread [33,34]. Specifically, IL-1β signaling is initiated upon its binding to the IL-1 receptor (IL-1R) and is subsequently propagated through the involvement of growth factors such as vascular endothelial growth factor (VEGF) and transforming growth factor beta (TGFβ), as well as transcription factors including STAT3 and NF-κB [29,35,36,37,38].

In the present study, we hypothesized that TEVs released by NSCLC cells bearing mutant EGFR and under IL-1β stimulation are implicated in promoting EMT in cancer cells by the activation of the fibronectin–α5β1 axis and in modulating the immune response by affecting immune checkpoint pathways (PD-1, CTLA-4, and FOXP3) and inflammatory mediators (IL-12, INF-γ, and TNF-α) in peripheral blood mononuclear cells (PBMCs) from lung cancer patients. In addition, TEVs isolated from osimertinib-resistant (OR) NSCLC cells were compared to those released by IL-1β-stimulated cells to identify new possible common biomarkers.

## 2. Results

### 2.1. Isolation and Characterization of sEVs from Sensitive and Resistant NSCLC Cells

**Figure 1:**
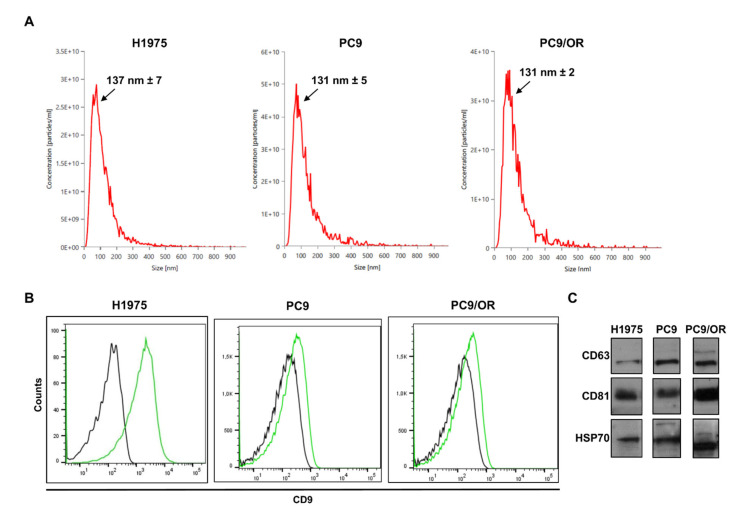
Size distribution and sEV markers in NSCLC TEVs. (**A**) The mean size and particle concentration of TEVs derived from H1975, PC9, and PC9/OR cells were assessed by NTA analysis. (**B**) FACS analysis of sEV markers in H1975, PC9, and PC9/OR TEVs (CD9: green curve; isotype control: black curve). (**C**) Western blot analysis of CD81, CD63, and HSP70 expression in H1975, PC9, and PC9/OR TEVs was performed using a comparable protein amount.

TEVs were isolated from H1975, PC9, and PC9/OR NSCLC cells and a size distribution analysis was performed by nanoparticle tracking analysis (NTA). In agreement with the minimal information for studies of EVs (guidelines MISEV 2023) [16], the registered size plots (Figure 1A) showed that most isolated sEVs reported a size <150 nm in diameter. In particular, the NTA results showed that vesicles isolated from all cell types were uniform in size and that the highest peak with slight variation was around 130 nm. Although the isolated sEVs may be not completely pure, NTA analysis did not show multiple distribution plots corresponding to the presence of a relevant sample contamination such as lipoproteins and protein aggregates with different sizes.

In addition, we further characterized our vesicles for the expression of sEV markers such as tetraspanins CD63 and CD81 and the chaperone protein HSP-70 [39]. The presence of the tetraspanin CD9 was also found and characterized by flow cytometry (Figure 1B). In addition, CD81 and HSP70 were found at high levels in each sample (Figure 1C) whereas CD63 was stronger expressed in the PC9 and PC9/OR TEVs than the H1975 TEVs containing a comparable amount of protein. In addition, we completed EV characterization, demonstrating that the calnexin, a known negative marker for sEVs, was selectively expressed only in H1975, PC9, and PC9/OR cells (Supplementary Figure S1).

### 2.2. TEV-Dependent Activation of EMT by Fibronectin Binding to α5β1 Integrin in NSCLCs

Fibronectin may be considered an EMT marker in cancer and we demonstrated that its receptor α5β1 has a relevant role in favoring cancer invasion in NSCLC cells [40]. Based on this evidence we aimed to test that TEVs were able to promote tumor invasiveness and progression by carrying fibronectin and thus activating α5β1 signaling in NSCLC cells.

**Figure 2:**
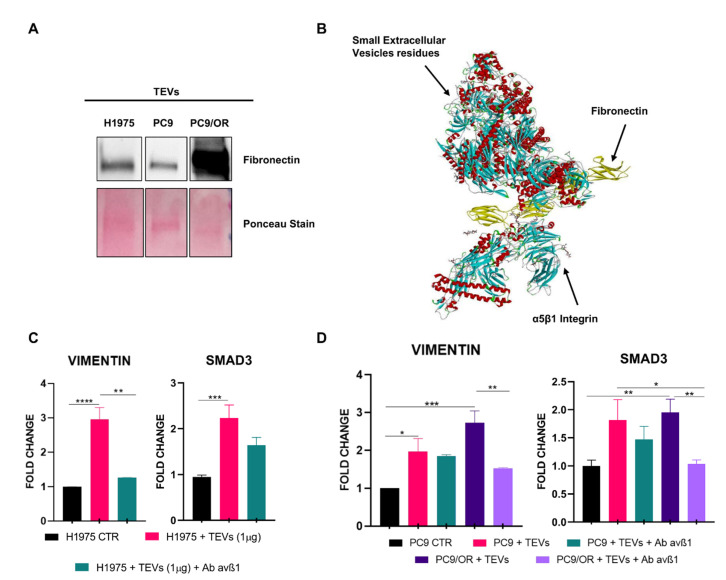
Fibronectin levels in NSCLC TEVs and EMT activation mediated by fibronectin interaction with α5β1 in NSCLC cells. (A) Western blot analysis of fibronectin levels contained in the isolated TEVs. (**B**) Three-dimensional representation of the top-ranked predicted docking pose between the integrin α5β1–fibronectin complex and the exosomal component, visualized using Discovery Studio Biovia. This pose shows the most likely interaction mode, highlighting the key binding sites and spatial arrangement of the two macromolecular complexes. (**C**,**D**) Effect of TEVs on EMT markers vimentin and SMAD3 in (**C**) H1975 and in (**D**) PC9 cells and in presence or absence of blocking anti-α5β1 antibody. Results are expressed as means ± SEM derived from two biological replicates calculated by the comparative method 2^−∆∆Ct^. Statistical significance: **** *p* < 0.0001, *** *p* < 0.001, ** *p* < 0.01, * *p* < 0.05.

Detectable levels of fibronectin were found in TEVs purified from H1975 and PC9 cells whereas the highest levels were found in the PC9/OR cells (Figure 2A). We previously demonstrated [40] that variable levels of α5β1 integrin were identified in NSCLC cells. In particular, both H1975 and PC9/OR cells exhibited 99% of an α5β1 integrin-positive cell population, whereas PC9 cells showed a markedly lower expression, around 65%. These findings suggest that α5β1 integrin expression on tumor cells may be related to distinct biological characteristics of the parental tumor cells, such as their drug resistance profile. In the interaction between sEVs containing fibronectin and the integrin α5β1 complex, specific amino acid residues are likely to be crucial for establishing and stabilizing the binding interface. This interaction could facilitate sEV adhesion and uptake by the target cells, thus eliciting signaling leading to migration and invasion. Within the fibronectin–integrin α5β1 complex (Figure 2B), residues such as ASP 1156C, VAL 1159C, and ARG 1243C are particularly significant, contributing to the overall binding strength. In particular, we focused our studies on the interaction of exosomes bearing fibronectin with integrin and found that, on the exosomal side, residues like GLU 173C, ILE 77K, and ARG 210K play active roles in engaging with the fibronectin–α5β1 complex, reinforcing their interaction. The residue pairs include 1156C–926K, 1241C–874K, and 1243C–922K, exemplifying some specific contacts that underpin the structural integrity and binding affinity of this complex. Furthermore, ARG 1360C from the fibronectin–integrin α5β1 complex and ARG 344K from the exosome are separated by less than 3 Å, highlighting another critical interaction point, indicative of a highly intimate binding interaction that may involve strong intermolecular forces. Similar short distances reflect the precise and robust nature of the binding interface, emphasizing the importance of these residues in stabilizing the exosome–fibronectin–integrin α5β1 complex. Remarkably, the HDOCK score for this pose is significantly negative, with a value of −257.68. This indicates a strong predicted binding affinity, underscoring the robust and favorable nature of the molecular interactions within this complex.

Next, to demonstrate the existence of a direct cause–effect link between fibronectin, carried by TEVs, and EMT activation mediated by an α5β1 receptor on NSCLCs, we tested the mRNA levels for vimentin and SMAD3 in the control and co-cultured cells with TEVs. In particular, this analysis was performed in parental H1975 and PC9 cells, incubated with TEVs in the presence or in the absence of blocking anti-α5β1antibodies (Figure 2C,D). As expected, we found that H1975 TEVs significantly upregulated the vimentin (black bars) and Smad 3 (gray bars) mRNA levels compared to the control (CTR) condition. Otherwise, blocking of fibronectin binding to α5β1 integrin, by a specific anti-α5β1 antibody, induced a clearcut reduction in the vimentin and SMAD3 levels. A similar effect was observed in the PC9 cells after co-culture with PC9 OR TEVs, whereas a milder effect was achieved in the same cells after exposure to PC9 TEVs. Notably, it is interesting to note that H1975 and PC9/OR TEVs, showing similar properties, were both released from cells with acquired resistance to EGFR TKIs of the first and third generation, respectively. Importantly, this EMT induction was significantly attenuated by the application of an α5β1-blocking antibody, underscoring the pivotal role of α5β1 integrin in mediating sEV-driven EMT and suggesting a potential therapeutic avenue to counteract TKI resistance in NSCLCs. Overall, these findings demonstrated that the TEV-mediated activation of the fibronectin–α5β1 axis in NSCLC cells may have a relevant role in promoting a transition towards a more aggressive phenotype.

### 2.3. Effect of IL-1β on Levels of Fibronectin and Cell Migration

**Figure 3:**
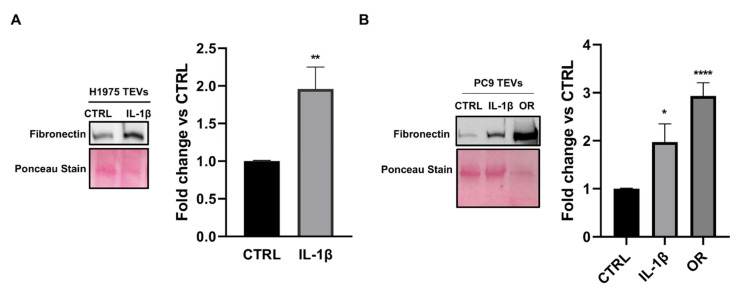
Levels of fibronectin in TEVs isolated from control and IL-1β stimulated cells. (**A**) TEVs were isolated from H1975, (**B**) PC9, and PC9/OR cells in basal condition or after treatment with 10 ng/mL IL-1β for 72 h. The total amount of protein in TEVs was calculated and Western blotting to assess fibronectin levels was performed using equal protein amounts for each sample. Quantitative analysis of gel bands by morpho-densitometric analysis using ImageJ software 1.53k was also performed. Data are expressed as relative protein levels of each sample compared to the corresponding control. Significant differences versus untreated were indicated with **** *p* < 0.0001, ** *p* < 0.01, * *p* < 0.05. At least three independent experiments were performed.

Based on the evidence that the pro-inflammatory cytokine IL-1β promotes cancer cell aggressiveness and may favor EMT pathway activation [41,42], we conducted Western blot analysis to determine whether IL-1β stimulation modulates the fibronectin levels in TEVs (Figure 3). We found that treatment with 10 ng/mL of such a cytokine for 72 h did not induce any significant effect on cell viability (Supplementary Figure S2) whereas such stimulation was able to increase the fibronectin levels in both H1975 and PC9 TEVs. However, the highest levels of fibronectin were found in PC9/OR TEVs. These findings well agree with the notion that EVs’ protein composition depends on the producer cell type and therefore the highest EMT signature score detected in PC9/OR cells may be related to the high fibronectin content of the vesicles [40]. Furthermore, these results were also confirmed by quantitative analysis of fibronectin bands detected in the different conditions (Figure 3A,B).

**Figure 4:**
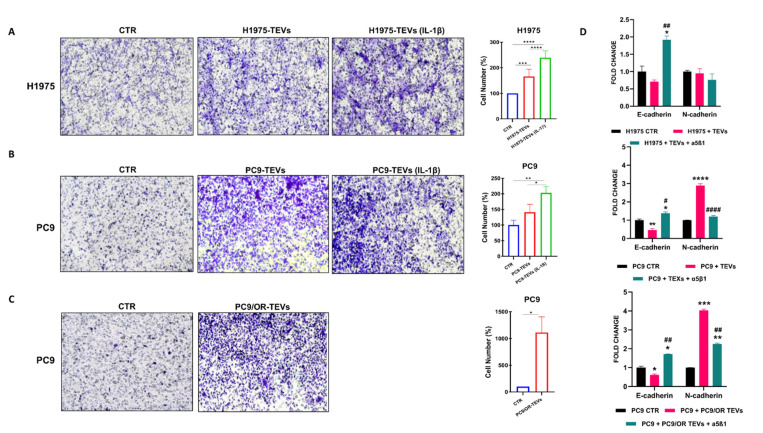
Effect of NSCLC TEVs from untreated and IL-1β-treated cells on cell migration. Migration assay was performed to detect the ability of TEVs, isolated from (**A**,**B**) 10 ng/mL IL-1β-untreated and treated H1975 and PC9 cells and (**C**) PC9/OR cells, in improving cell motility. Three biological replicates were performed and quantitative analysis of migrated cells was assessed and expressed as percentage, considering the untreated control cells as 100%. (**D**) RT-PCR analysis of the TEV-mediated modulation of E- and N-cadherin in H1975 and in PC9 cells and in presence or absence of blocking anti-α5β1 antibody. Statistical significance is indicated as follows: **** *p* < 0.0001, *** *p* < 0.001, ** *p* < 0.01, * *p* < 0.05; ^####^ *p* < 0.0001, ^##^ *p* < 0.01, ^#^ *p* < 0.05.

Additionally, we aimed to assess whether the detected high levels of sEV-associated fibronectin correlate with the enhanced migratory capacity of the cancer cells from which these TEVs originate (Figure 4). As previously described, it is also well known that high levels of fibronectin and EMT signaling activation in cancer are related to cell migration and invasion. Based on this evidence, we demonstrated that NSCLC TEVs were able to increase cancer cell migration and that this effect was amplified by IL-1β stimulation. Figure 4 illustrates the results of a cell migration assay, where TEVs isolated from the three selected cell lines (H1975, PC9, and PC9/OR) were placed in the lower chamber of a transwell system and the ability of cells to migrate through the transwell membrane was assessed after 24 h. Migrated cells through the transwell membrane for each condition were visualized and counted. For both the H1975 and PC9 cells, cell migration under the serum-free (SF) condition was minimal. We found that treatment with TEVs from both cell lines improved migration compared to the SF condition and that such improvement was also significantly increased by TEVs isolated upon IL-1β cell stimulation (Figure 4A,B). Similarly, TEVs derived from PC9/OR cells elicited the most pronounced and significant enhancement of PC9 cell migration, confirming the potent pro-migratory effect of EVs originating from resistant cells.

In agreement with these findings, we also tested the effect of TEVs on modulating the E-cadherin and N-cadherin mRNA levels. It is well known that E-cadherin downregulation and N-cadherin upregulation are related to a reduction in cell-to-cell adhesion, thus resulting in increased cellular motility and migration ability. Interestingly, we found that H1975, PC9, and PC9/OR TEVs were able to decrease the E-cadherin mRNA levels and that cell incubation with an α5β1-blocking antibody may inhibit such a process (Figure 4D). Furthermore, we found an increase in the N-cadherin mRNA levels in cells after incubation with PC9 and PC9/OR TEVs and that treatment with an α5β1-blocking antibody may hinder such an improvement. No modulation of N-cadherin was found in H1975 cells.

### 2.4. Effect of IL-1β on PD-L1 Levels in TEVs Isolated from NSCLC Cells and PCR Analysis of sEV-Dependent Modulation of Immune Genes in PBMCs

**Figure 5:**
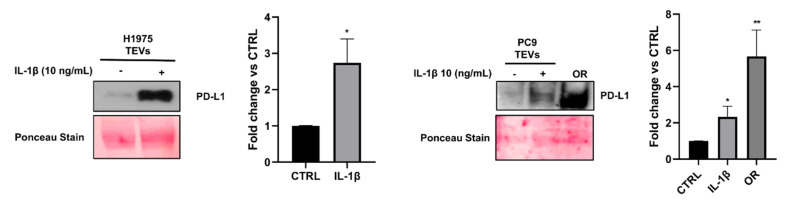
Levels of PD-L1 in TEVs isolated from sensitive and resistant NSCLC cells and effect of IL-1β on protein expression. TEVs were isolated from H1975, PC9, and PC9/OR cells in basal condition or after treatment with 10 ng/mL IL-1β for 72 h. The total amount of protein was assessed in EVs and Western blotting analysis for PD-L1 detection was carried out using equal protein amounts for each sample. Quantitative analysis of protein levels was also performed. Data were expressed as relative protein levels of each sample compared to the corresponding control. Significant differences versus untreated were indicated with ** *p* < 0.01, * *p* < 0.05. At least three independent experiments were performed.

In order to demonstrate whether NSCLC-derived TEVs were able to mediate cancer progression by modulating the immune response, the levels of the immune checkpoint PD-L1 in TEVs isolated from unstimulated and IL-1β-stimulated cells as well as in OR cells were assessed (Figure 5). We found that stimulation with 10 ng/mL IL-1β for 72 h caused an upregulation of PD-L1 expression compared to the control cells. As expected, the highest level of this protein was found in OR TEVs, reflecting the highest tumor aggressive phenotype related to immune evasion. Furthermore, we also assessed PD-L1 expression in both sensitive and OR cells (Supplementary Figure S3). We found that similarly to TEVs, the PD-L1 levels were higher in resistant cells and after IL-1β stimulation than the control cells, thus suggesting that TEVs and cancer cells, from which they are released, may synergistically contribute to tumor immune evasion.

**Figure 6:**
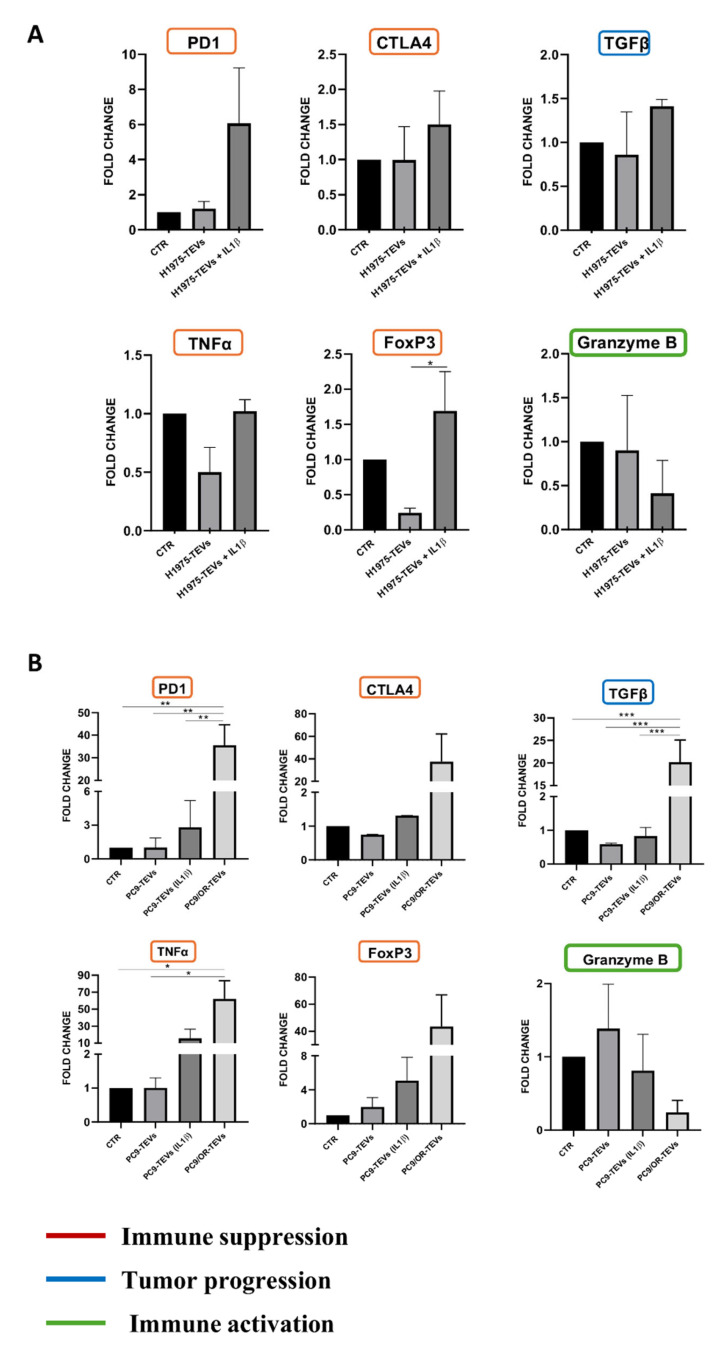
Relative expression levels of immune genes and cytokines (TNFɑ and TGF-β) in PBMCs derived from lung cancer patients after treatment with TEVs. TEVs derived from H1975 (**A**), PC9, and PC9/OR (**B**) cell lines untreated and treated with IL-1β and containing comparable amounts of proteins (25 µg) were incubated with PBMCs from lung cancer patients and gene expression was analyzed using RT-PCR. The data are presented as bar graphs comparing three conditions: PBMC CTR (control PBMCs), PBMC TEVs (PBMCs treated with TEVs), PBMC TEVs IL-1β (PBMCs treated with TEVs from H1975 and PC9 cells treated with 10 ng/mL IL-1β), and PBMC TEVs OR (PBMCs treated with TEVs from PC9/OR cells). Normalized expression data are presented as the mean ± S.E.M. derived from two biological replicates via the comparative method 2^−ΔΔCt^ (reference gene 18S). Statistical significance is indicated as follows: * (*p* < 0.05), ** (*p* < 0.01), *** (*p* < 0.001). FC: Fold change.

To gain insights into the role of NSCLC TEVs in modulating immune cell gene expression and sensitivity to IL-1β, we isolated PBMCs from lung cancer patients and co-cultured them with H1975, PC9, and PC9/OR TEVs prior to mRNA isolation. In particular, Figure 6 shows that H1975 TEVs isolated from the IL-1β-treated cells significantly upregulated the mRNA levels for immune checkpoint PD-1 and CTLA-4 along with the FOXP3 transcription factor involved in immune surveillance reduction. Similarly, an increase in the PD-1 protein levels was also detected in PBMCs co-cultured with TEVs, thus providing strong evidence of functional immunomodulation (Supplementary Figure S4).

In agreement with these findings, we also observed concurrent upregulation of transforming growth factor beta (TGF-β), which is reported to play a critical role in immune suppression mediated by FOXP3+ regulatory T cells. After TEV co-culture with PBMCs, we also observed an increase in TNF-ɑ, one of the most powerful pro-tumoral cytokines in many cancer types [43]. A reduction in Granzyme B, a serine protease mainly secreted by NK and cytotoxic T cells and involved in anti-tumor T cell response [44,45], was also highlighted. Similar results were also obtained after PBMC co-culturing with PC9 TEVs for 72 h. In particular, PC9 TEVs isolated from IL-1β-treated cells were able to increase the PBMCs’ mRNA levels for PD-1, CTLA-4, FOXP3+, and TNF-ɑ and reduce Granzyme B mRNA levels compared to that observed in PBMCs co-cultured with TEVs isolated from the control cells. Similar results were also obtained after immune cells’ incubation with PC9/OR TEVs that were able to induce the strongest modulatory effect on gene expression.

**Figure 7:**
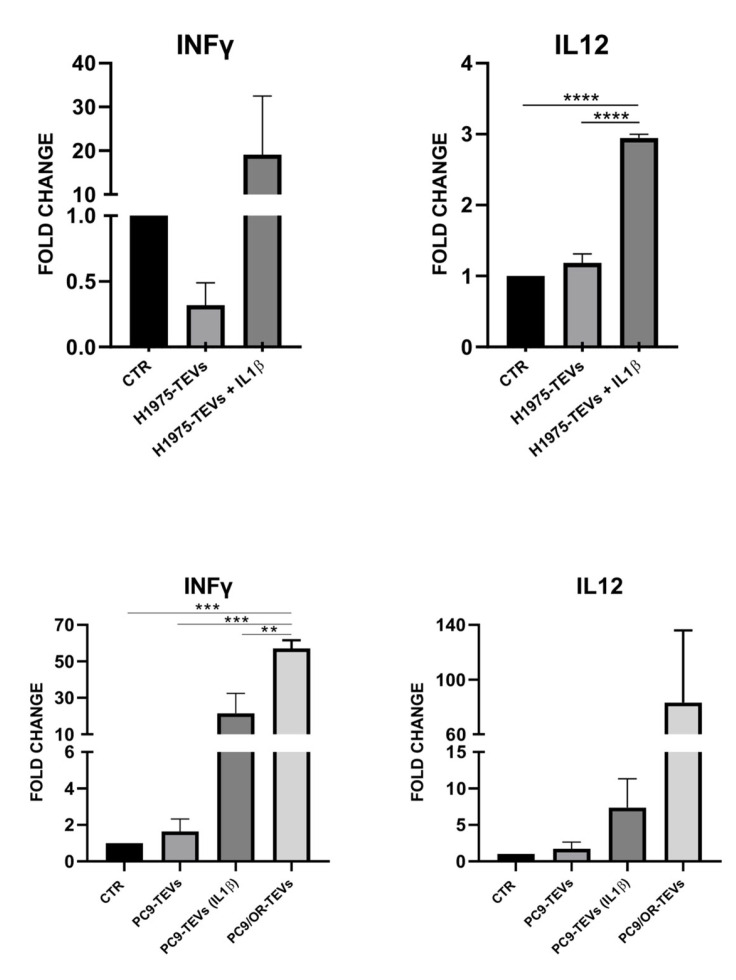
Levels of pro-inflammatory cytokines IFN-γ and IL-12 in PBMCs before and after co-culturing with H1975, PC9, and PC9/OR TEVs. NSCLC TEVs derived from control, IL-1β-treated, and OR cells were incubated with PBMCs and gene expression was analyzed using RT-PCR. The data are presented as bar graphs comparing three conditions: PBMC CTR (control PBMCs), PBMC TEVs (PBMCs treated with TEVs), PBMC TEVs IL-1β (PBMCs treated with TEVs from H1975 and PC9 cells treated with 10 ng/mL IL-1β), and PBMCs TEVs OR (PBMCs treated with TEVs from PC9/OR cells). Normalized expression data are presented as the mean ± S.E.M. derived from two biological replicates via the comparative method 2^−ΔΔCt^ (reference gene 18S). Statistical significance is indicated as follows: **** *p* < 0.0001, *** *p* < 0.001, ** *p* < 0.01. FC: Fold change.

Furthermore, in addition to such evidence, we also found a concomitant upregulation of pro-inflammatory cytokines IFN-γ and IL-12 in PBMCs co-cultured with NSCLC TEVs from OR and IL-1β-stimulated cells (Figure 7). Recent studies recognized some clearcut pro-tumoral effects of IFN-γ that may be upregulated by IL-12 and in a context-dependent manner [46,47]. In particular, it was reported that IFN-γ may drive the upregulation of PD-L1 in cancer cells and the TME’s cells as well as stimulate more IL-12 secretion, thus activating a positive feedback loop of IFN-γ. This mechanism has been shown for both tumor and inflammation sites [48].

## 3. Discussion

Based on their biogenesis and composition, EVs closely resemble the genotypic and phenotypic signature of the originating cells and transfer biological information in recipient cells. These shuttles have fundamental roles in both physiological and pathological processes and here we explore their function in cancer progression and resistance to therapy by EMT activation and immune modulation in a specific subtype of NSCLC, the EGFR mutant one, that is known to develop features of EMT and immune evasion under resistance to targeted agents [46,49,50]. Noticeably, these effects are enhanced by IL-1β, a cytokine released by monocytes, macrophages, and neutrophils during chronic inflammation that is associated with bad prognosis and tumor progression in multiple solid tumors [47]. In this context, the novel findings of the present study demonstrate that EGFR mutant NSCLC cancer cells stimulated with IL-1β may release sEVs containing factors that promote epithelial–mesenchymal transition (EMT) in recipient cells, the downregulation of anti-tumor immune response, and the survival of circulating metastatic cells. In particular, we demonstrated that TEVs released by IL-1β-stimulated EGFR mutant NSCLC cells are enriched with fibronectin, similarly to the ones released from the EGFR-resistant model PC9/OR. These findings suggested a novel mechanism involved in EGFR resistance: in EGFR mutant NSCLCs, inflammation may be a driver for the TEV-mediated activation of the fibronectin–α5β1 axis. This hypothesis is supported by our results that showed in NSCLC cells an sEV-dependent upregulation of EMT-related genes (vimentin, SMAD3) and cadherin switch (E-cadherin reduction and N-cadherin increase) that occurred via an autocrine mechanism and that is hindered by blocking integrin α5β1 signaling. These results suggest that, within the heterogeneous population of drug-resistant cancer cells, a selective drug pressure may favor, in resistant clones, the synthesis and release of TEVs able to confer to recipient cells the expression of EMT signature markers, known to be linked to stemness properties, resistant phenotypes, and improved migration ability. Furthermore, the presence of high fibronectin levels in TEVs after IL-1β stimulation or after the occurrence of drug resistance may induce EMT in stromal cells with the formation of a pre-metastatic niche [51].

In our study, we also showed that IL-1β stimulation induced the release of PD-L1-enriched TEVs, with consequent implications through paracrine mechanisms on TME response and immune surveillance mechanisms against circulating tumor cells. These data are also supported by the analysis of mRNAs isolated from lung patients’ PBMCs before and after TEV co-culturing that showed a concomitant reduction in immune genes and upregulation of TGF-β involved in tumor progression.

Also, our findings demonstrated that, paralleling their effects on cell migration and EMT modulation, the greatest suppression of immune cell function was observed in PBMCs stimulated with OR TEVs. These results in NSCLCs suggest that elevated levels of the pro-inflammatory cytokine IL-1β in the extracellular environment may endow TEVs with biological properties akin to those of OR TEVs produced under selective drug pressures. This implies that IL-1β can modulate the immunosuppressive and pro-tumorigenic functions of TEVs, contributing to tumor progression and immune evasion.

Globally, our findings suggest that TEVs profoundly alter immune cell phenotypes, redirecting the immune response towards an immunosuppressive status, thereby facilitating tumor progression. Notably, it is also known that in both tumor and inflamed environments, there exists a positive feedback loop of IFN-γ, which may also stimulate APCs to secrete more IL-12, which triggers the re-activation of the IFN-γ production cycle [48]. We speculate that the activation of gene transcription serves as a surrogate marker for the protein expression of these immunological markers, which tend to be activated simultaneously. We are aware that further studies will be needed to elucidate the specific roles of these mediators, based on proteomic assays.

Future research should aim to elucidate the precise molecular mechanisms driving these TEV-mediated effects and evaluate the therapeutic potential of targeting EVs’ biogenesis, secretion, or function in lung cancer patients.

In conclusion, increased inflammation, exemplified by elevated IL-1β levels in our study, may act synergistically with drug resistance mechanisms to promote treatment failure and tumor recurrence, highlighting inflammation as a critical cofactor in cancer progression and as a new potential mechanism to target in EGFR mutant-resistant models.

## 4. Materials and Methods

### 4.1. Cell Culture

Two EGFR-mutated NSCLC H1975 and PC9 cell lines were obtained from and authenticated by the American Type Culture Collection (ATCC, Manassas, VA, USA). In addition, PC9 cells were exposed to continuous osimertinib stimulation in order to establish the corresponding osimertinib-resistant (OR) cell line as previously described [40]. All cell lines were grown in RPMI 1640 (Gibco) medium supplemented with 10% fetal bovine serum (FBS), 100 IU/mL penicillin, and 50 µg/mL streptomycin in a humidified incubator in 5% CO_2_ at 37 °C. PC9/OR cells are always maintained with osimertinib at a dose lower than their IC50, except during specific experiments.

### 4.2. sEV Isolation from Cell Lines

Before isolating sEVs, all cell lines were maintained for 72 h in EV-free medium with 10% of FBS, previously ultracentrifuged at 100,000× *g* at 4 °C for 16–18 h and filtered using a membrane with 0.22 µm pore size. H1975, PC9, and PC9/OR TEVs were isolated following a protocol previously described [52] from unstimulated and 10 ng/mL IL-1β-stimulated cells. Briefly, cell culture media were collected and subjected to a first centrifugation process at 300× *g* for 10 min. Subsequently, the supernatant was subjected to a second centrifugation process at 2000× *g* for 10 min to remove dead cells. Then, the medium containing EVs was transferred into specific tubes (Ultra-clear tubes, Beckman, Brea, CA, USA) and centrifuged at 10,000× *g* for 30 min. The supernatant containing EVs was filtered through a 0.22 μm filter (Millipore) and transferred to new tubes and centrifuged two times at 4 °C and 100,000× *g* for 70 min. The pellets (containing the sEVs) were resuspended in cold sterile PBS and samples were stored at −80 °C or immediately used.

### 4.3. Immunoblotting Analysis

The protein content from TEVs was determined by Bradford assay (Bio-Rad, Hercules, CA, USA) as previously described [52]. Western blot analysis of proteins from different TEV preparations and conditions was performed using a standard procedure. TEV samples containing comparable amounts of proteins (2–10 μg) were resuspended in LDS reducing sample buffer (Thermo Fisher B0007, Waltham, MA, USA), mixed, and boiled at 100 °C for 10′. Samples were resolved by SDS-PAGE gels and transferred onto PVDF membranes (Millipore, Burlington, MA, USA). After blocking membranes for 90 min at room temperature, they were probed overnight with primary antibodies. Horseradish peroxidase-linked anti-rabbit (BioRad) and anti-mouse (BioRad) antibodies were used as secondary antibodies. Antibodies used for Western blotting included monoclonal anti-HSP70 (sc-24, Santa Cruz Biotechnology, Dallas, TX, USA), anti-CD81 (sc166029, Santa Cruz Biotechnology) and anti-CD63 (sc-15363, Santa Cruz Biotechnology), PD-L1 (13684S, Cell Signaling) and anti-Fibronectin (sc-271098 Santa Cruz Biotechnology), and monoclonal anti-Calnexin (SPA-860, Stressgen Biotechnologies, Victoria, BC, Canada). A commercially available ECL kit (Advansta, San Jose, CA, USA) was used to reveal protein bands that were quantified by morpho-densitometric analysis using ImageJ software version Java8 (NIH, Bethesda, MD, USA).

### 4.4. Flow Cytometry

For staining, 2.1 × 10^13^ particles of TEVs (50 μL) were mixed with 40 μL of filtered PBS containing 0.4 μg of BUV496 Mouse Anti-Human CD9 (BD Biosciences, Franklin Lakes, NJ, USA) or anti-CD9 APC-A750 mouse monoclonal antibody (Beckman Coulter), isotype control antibody IgG1 APC-A750 (provided by Beckman Coulter), or BUV496 Mouse Anti-Human IgG (BD Biosciences) at 4 °C for 30 min in the dark. For the removal of unbound antibodies, size-exclusion chromatography (SEC) columns were employed (iZON qEV original columns SP1, United Kingdom) [53]. Samples containing stained EVs, and appropriate controls, were diluted up to 0.5 mL with PBS and analyzed with a BD FACS ARIA III (Becton & Dickinson, Mountain View, CA, USA). For the detection of small particle populations, single or dual thresholds were used to set up the instrument. The unstained control was used to set the quadrant gating. Registered data were elaborated using BD FACSDiva™8.0 Software ((Becton & Dickinson, Mountain View, CA, USA). At least three independent experiments were performed.

### 4.5. Nanoparticle Tracking Analysis (NTA)

Nanoparticle tracking analysis (NTA) of TEVs from NSCLC H1975, PC9, and PC9/OR cells was performed to determine EVs’ size distribution plots, particle concentration, and purity of preparation using a Malvern Panalytical NanoSight Pro (Malvern Instruments, Amesbury, UK) equipped with a 488 nm laser. As previously described [52], a high-sensitivity sCMOS (USB-3) camera was used to acquire five videos with software-optimized settings and a sample flow rate between 5 and 15 μL/min. Registered data were analyzed using NS Xplorer software v1.1.0.6.

### 4.6. MTS Assay

IL-1*β*-induced toxicity was assessed by using the MTS assay (Cell Counting Kit-8). Briefly, cells were seeded in 96-well flat-bottomed plates at a density of 3000 cells/well and treated for 72 h with 10 ng/mL IL-1β for 72 h. Viable cell number was determined spectrophotometrically at 450 nm and expressed as the cell percentage considering the untreated control as 100%.

### 4.7. Cell Migration Assay

Cells (~150,000) were resuspended in 100 μL RPMI supplemented with 1% FBS and seeded onto the top of transwell inserts placed in a 24-well plate. Cell migration was stimulated by supplementing serum-free cell culture medium, in the transwell lower compartment, with sEVs containing 1 µg proteins, as a chemoattractant, for 24 h. After incubation, cells were fixed with 70% ethanol for 10 min and stained with 0.2% crystal violet for a further 10 min and counted using an inverted microscope. Non-migrated cells, on the top of the membrane, were gently removed with a cotton-tipped applicator. Colored migrated cells were visualized with an inverted high-resolution microscope equipped with a 20X and 10X lens (ECLIPSE Ti2, Nikon, Japan). Five fields for each membrane were photographed and the number of invading cells were counted and determined. Three independent experiments were performed and pooled together.

### 4.8. PBMCs Isolation

Human samples were collected after obtaining a written informed consensus from patients in accordance with the Declaration of Helsinki. The protocol for the use of these samples for research purposes was approved by the Ethics Committee of the University of Campania “Luigi Vanvitelli”, Naples (n. 280 on 16 May 2020). Peripheral blood mononuclear cells (PBMCs) were isolated from NSCLC patients using a density gradient centrifugation method established in our previous work [54]. Briefly, 6–7 mL of blood mixed with the anticoagulant EDTA was drawn into collection tubes and gently layered over 4 mL of Ficoll in a 15 mL centrifuge tube. The samples were then centrifuged at 2000 rpm for 20 min at room temperature, ensuring no acceleration or braking was used. This process separated the blood components according to their densities. The layer enriched with leukocytes and platelets was carefully collected and transferred to fresh tubes, followed by a second centrifugation at 1600 rpm for 15 min. To eliminate any remaining red blood cells, 1 mL of RBC lysis buffer was added, and the mixture was centrifuged again at 1600 rpm for 12 min. The isolated PBMCs were then resuspended in 10 mL of RPMI medium for subsequent analysis.

### 4.9. TEV Co-Culture with NSCLC Cells and PBMCs from Lung Cancer Patients

To demonstrate that EMT activation in NSCLCs may be promoted by the interaction of TEVs with α5β1 on cell membranes, H1975 and PC9 cells (~100,000) were plated and grown in EV-free medium at 10% of FBS for 3 days. After this time, cells were incubated for 1 h with 0.5 µg/mL of blocking antibody recognizing α5β1 (Millipore, cat. MAB2514) and then TEVs containing 1 µg protein were added. After 72 h of co-culture, cells were centrifuged at 1600 rpm for 12 min and the supernatant was discarded. The pellets were immediately processed or stored at −80 °C until RNA extraction.

In parallel, to evaluate the effect of TEVs on immune genes and cytokine expression in PBMCs, H1975, PC9, and PC9/OR TEVs containing comparable amounts of proteins (25 µg) were added to 500,000 immune cells. After 72 h of co-culture at 37 °C, PBMCs were centrifuged at 1600 rpm for 12 min and the supernatant was discarded. The samples were washed with PBS and centrifuged again to recover the resultant cell pellets that were immediately processed or stored at −80 °C until RNA extraction.

### 4.10. RNA Extraction and cDNA Synthesis from NSCLCs and PBMCs

Trizol reagent was added to cell pellets derived from both NSCLC cells and PBMCs. The pellets were resuspended and the samples were centrifuged at 12,000 rpm for 10 min. Subsequently, 100 µL of chloroform was added to each sample, and the tubes were vigorously shaken to promote phase separation, followed by centrifugation at 15,000 rpm for 15 min. The aqueous phase, containing the RNA, was carefully transferred to fresh tubes, and isopropanol was added to precipitate the RNA. The tubes were then stored at −80 °C over the weekend. Upon removal, samples were centrifuged at 12,000 rpm for 15 min. The supernatant was discarded, and the resulting RNA pellet was washed with 1 mL of 70% ethanol. This washing step was repeated several times, each time centrifuging at 15,000 rpm for 5 min at 4 °C. After the final washing, the supernatant was removed, and the tubes were left to air-dry under a chemical hood for 30 min. The RNA pellet was then dissolved in 50 µL of RNase-free water. RNA purity and concentration were determined using a Nanodrop 2000 spectrophotometer (Thermo Fisher Scientific, Waltham, MA, USA), with absorbance measured at 260/280 nm. For cDNA synthesis, 1 µg of total RNA from each sample was reverse transcribed using the SensiFAST cDNA Synthesis Kit (BIO-65053, Meridian Bioscience, Memphis, TN, USA). The reverse transcription protocol consisted of incubation at 25 °C for 10 min, 42 °C for 15 min, and a final step at 85 °C for 5 min.

### 4.11. mRNA Gene Expression Analysis by RT-PCR

The mRNA expression levels of *vimentin*, *SMAD3*, *N-cadherin*, *E-cadherin*, *PD-1*, *CTLA-4*, *INF-ɣ*, *INF-β*, *TNF-α*, *FOXP-3*, *IL-2*, *TGF-β*, *IL-12*, *GRANZYME B*, and *TGF-βR* genes were assessed through qRT-PCR using a QuantStudio 7-Flex instrument (Applied Biosystems by Life Technologies, Monza, Italy) and the SensiFAST SYBR Hi-ROX Kit (BIO-92005, Meridian Bioscience, Memphis, TN, USA) at the following conditions: 50 °C for 2 min (stage 1) followed by a denaturation step at 95 °C for 10 min (stage 2) and then 40 cycles at 95 °C for 15 s and 60 °C for 1 min (stage 3). All samples underwent duplicate runs in 20 µL reactions. The relative expression of genes was determined by normalization to 18S, serving as an internal control gene. Relative gene expression values were calculated using the 2^−ΔCt^ or 2^−ΔΔCt^ method. Supplementary Table S1 shows the list of primer sequences used for qRT-PCR. Changes in mRNA levels were normalized by referring to the expression of housekeeping genes (18S). Data are expressed as means ± SEM derived from two biological replicates by the comparative method 2^−∆∆Ct^ towards untreated PBMCs.

### 4.12. Molecular Docking Simulation of Integrin α5β1 Binding to Fibronectin Exposed on TEVs

We utilized HDOCK to perform docking simulations between the fibronectin–integrin α5β1 complex and the exosomal component (Figure 2B, Supplementary Table S2). This software is particularly effective for modeling biomacromolecule interactions in silico, offering dimensionless docking scores where more negative values indicate greater stability and favorability of the predicted poses (HDOCK score: −257.68). The fibronectin–integrin α5β1 structure was sourced from the Protein Data Bank (PDB ID: 7NWL), while the exosomal component (PDB ID: 6D6Q) was refined by Discovery Studio software (Discovery Studio DS 2021; Accelrys, San Diego, CA, USA) before analysis. The more favorable pose is depicted in Figure 2B.

### 4.13. Statistical Analysis

Data are presented as the means ± standard deviations (SD) or standard errors (SEs). The Western blot signals were quantified by morpho-densitometric analysis using ImageJ software version Java8 (NIH, Bethesda, MD, USA). Briefly, the product of the area and optical density of each band were determined and normalized to the same parameter derived from the control. The data were expressed as the relative protein levels of each sample compared with those of the corresponding control. *p*-values were determined using unpaired Student’s *t*-tests or ordinary one-way ANOVA (GraphPad Prism software, version 8). In particular, for groups with normal distributions and equal variances, a parametric unpaired *t*-test was used. When three or more groups were compared, a one-way ANOVA test was used. A *p*-value lower than 0.05 was considered the threshold for statistical significance.

## 5. Conclusions

The differential effects observed among TEVs released from various cancer cell lines underscore the complexity of extracellular vesicles’ communication within the TME. Our study indicates that TEVs produced following IL-1β signaling activation, as well as those from both sensitive and resistant EGFR TKI-treated cells, contribute not only to tumor progression, through enhanced cell migration and EMT modulation, but also to the alterations in immune cell populations, potentially facilitating immune evasion. These findings align with emerging evidence that TEVs modulate the TME by influencing stromal and immune cells, promoting pro-tumorigenic inflammation, immune suppression, and metastatic niche formation.

Overall, this study advances our understanding of TEV release as a coordinated process orchestrating cancer progression. It emphasizes the necessity of incorporating EV-mediated intercellular communication into comprehensive cancer treatment strategies, potentially improving therapeutic efficacy by disrupting the multifaceted roles of extracellular vesicles in tumor growth, immune modulation, and drug resistance.

Specifically, in the heterogeneous context of EGFR mutant NSCLCs with a resistant phenotype, further study on sEV regulation may represent a big opportunity to modulate the known features of drug resistance based on EMT, immune evasion, and stemness properties.

## Data Availability

The majority of data related to the presented results are included in the Materials and Methods section of this paper.

## Supplementary Materials

The following supporting information can be downloaded at: https://www.mdpi.com/article/10.3390/ijms26146825/s1.

## Abbreviations

The following abbreviations are used in this manuscript:

IL-1β	Interleukin-1 beta
TME	Tumor microenvironment
TEVs	Tumor-derived small extracellular vesicles
OR	Osimertinib-resistant
NSCLC	Non-small-cell lung cancer
PD-L1	Programmed death-ligand 1
SMAD3	Mothers against decapentaplegic homolog 3
PBMCs	Peripheral blood mononuclear cells
CTLA-4	Cytotoxic T-lymphocyte-associated protein 4
FOXP3	Forkhead box P3
TNF-α	Tumor necrosis factor
IL-12	Interleukin-12
INF-γ	Interferon gamma
EMT	Epithelial–mesenchymal transition
EVs	Extracellular vesicles
sEVs	Small extracellular vesicles
EXs	Exosomes
ApoEVs	Apoptotic bodies
MVs	Microvesicles
MVBs	Multivesicular bodies
IL-1R	Interleukin-1 receptor
VEGF	Vascular endothelial growth factor
TGF-β	Transforming growth factor beta
STAT-3	Signal transducer and activator of transcription 3
NF-κB	Nuclear factor kappa-light-chain-enhancer of activated B cells
EGFR	Epidermal growth factor receptor
PD-1	Programmed cell death protein 1
FBS	Fetal bovine serum
LDS	Lithium dodecyl sulfate
SDS-PAGE	Sodium dodecyl sulfate polyacrylamide gel electrophoresis
PVDF	Polyvinylidene fluoride
HSP-70	Heat shock protein 70
SEC	Size-exclusion chromatography
NTA	Nanoparticle tracking analysis
EDTA	Ethylenediaminetetraacetic acid
RBC	Red blood cells
SD	Standard deviation
SE	Standard error
MISEV	Minimal Information for Studies of Extracellular Vesicles
ASP	Aspartic acid
VAL	Valine
ARG	Arginine
CTR	Control
TKIs	Tyrosine kinase inhibitors
SF	Serum-free
APCs	Antigen-presenting cells
