# Supplementary material for: Effect of IL-1β on NSCLC-Derived Small Extracellular Vesicles as Actors in Mediating Cancer Progression and Evading Immune System
Source: Int J Mol Sci. 2025 Jul 16;26(14):6825. doi: 10.3390/ijms26146825 (PMC12295926; doi:10.3390/ijms26146825)
Supplement: Supplementary file 1 [file ijms-26-06825-s001.zip › Supplementary_materials Rev2.pdf]

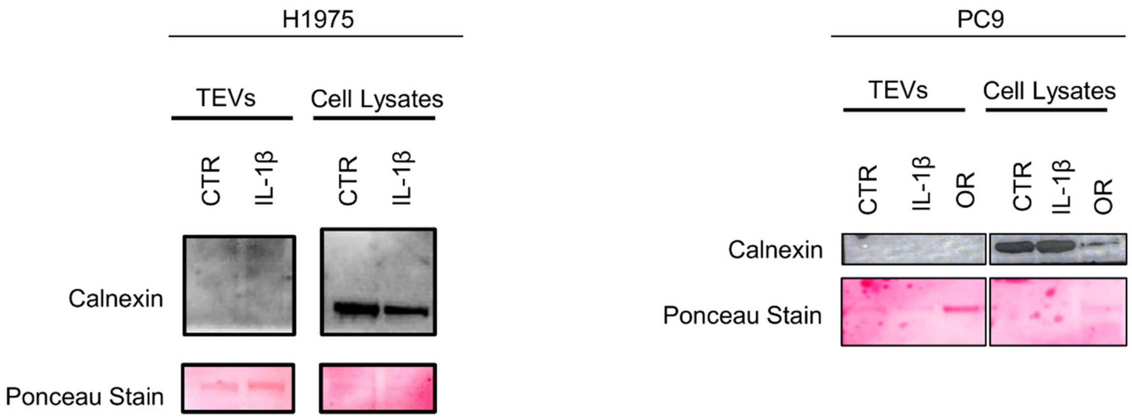

Supplementary Figure 1. Western blot analysis of calnexin expression in H1975, PC9 and PC9/OR TEVs and corresponding whole cell lysates. Calnexin was used as a negative exosomal marker and Ponceau S staining was performed to ensure protein loading.

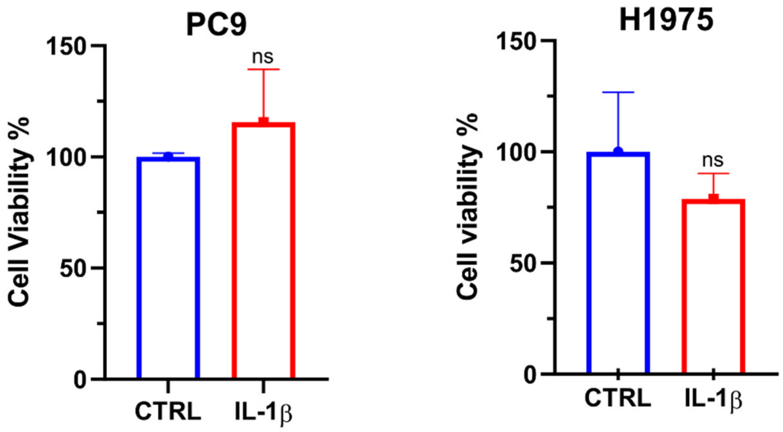

Supplementary Figure 2. Effect of 10 ng/mL IL-1β treatment for 72h on NSCLC cells viability. No statistically significant difference was observed between treated and untreated cells.

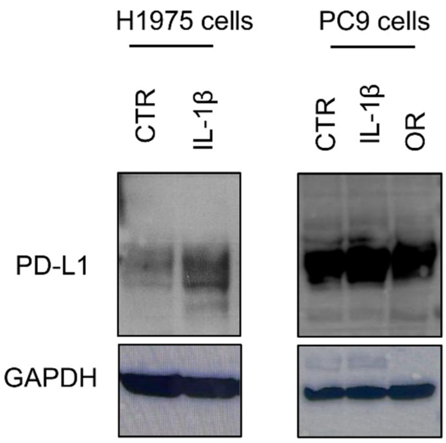

Supplementary Figure 3. Western blot analysis of PD-L1 expression in H1975, PC9 and PC9/OR whole cell lysates. GAPDH was used to ensure equal protein loading.

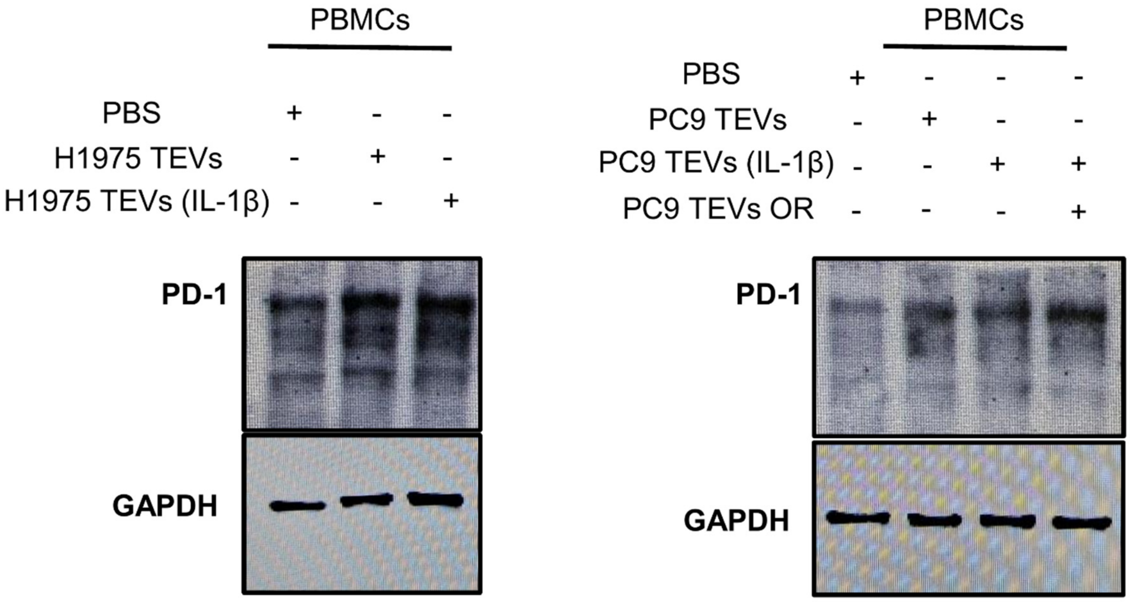

Supplementary Figure 4. Western blot analysis of PD-1 expression in PBMCs co-cultured with H1975, PC9 and PC9/OR TEVs. GAPDH was used to ensure equal protein loading.

Gene	*Forward sequence*	*Reverse sequence*
**18s**	5'-CGCCGCTAGAGGTGAAATTC-3'	3' -CTTTCGCTCTGGTCCGTCTT-5'
**PD-1**	5'-AAGGCGCAGATCAAAGAGAGCC-3'	3'-CAACCACCAGGGTTTGGAACTG-5'

**CTLA-4**	5'-AAGGTGGAGCTCATGTACCC-3'	3'-TCTGGGTTCCGTTGCCTATG-5'
**INF-γ**	5'-ATGGCTGAACTGTCGCAAG-3'	3'- TGCAGGCAGGACAACCATT-5'
**INF-β**	5'-GGAAAAGCAAGAGGAAAGATTGAC-3'	3'-CCACCATCCAGGCGTAGC-5'
**TNF-α**	5'-TCTGTCTGCTGCACTTTGGAGTGA-3’	3' -TTGAGGGTTTGCTACAACATGGGC-5’
**FOXP-3**	5'-AGACCCCTGTGCICCAAGTG-3'	3'-CAGACTCCATTIGCCAGCAG-5'
**TGF-β**	5'-TACCTGAACCCGTGTTGCTCTC-3'	3'-GTTGCTGAGGTATCGCCAGGAA-5'
**IL-12**	5'-TTTATGATGGCCCTGTGCCT-3'	3’-GGTCTTGAACTCCACCTGGTA-5'
**Granzyme B**	5'-TGCATCTTGGTCCGATACTCT-3'	3'-GGCTTCCAGAATCTCCATTGC-5'
**N-cadherin**	5'-CCTCCAGAGTTTACTGCCATGAC-3'	3’-GTAGGATCICCGCCACTGATTC-5'
**E-cadherin**	5’-ATTTTTCCCTCGACACCCGAT-3’	3’-AGAACCAGATGCGGACCCT-5’
**Vimentin**	5'-AGGCAAAGCAGGAGTCCACTGA-3’	3’-ATCTGGCGTTCCAGGGACTCAT-5'
**SMAD3**	5'-TGAGGCTGTCTACCAGTTGACC-3'	3’-GTGAGGACCTTGTCAAGCCACT-5’

Supplementary Table 1. List of primer sequences used for qRT-PCR.
